# Neighborhood level risk factors for type 1 diabetes in youth: the SEARCH case-control study

**DOI:** 10.1186/1476-072X-11-1

**Published:** 2012-01-09

**Authors:** Angela D Liese, Robin C Puett, Archana P Lamichhane, Michele D Nichols, Dana Dabelea, Andrew B Lawson, Dwayne E Porter, James D Hibbert, Ralph B D'Agostino, Elizabeth J Mayer-Davis

**Affiliations:** 1 Department of Epidemiology and Biostatistics and Center for Research in Nutrition and Health Disparities, Arnold School of Public Health, University of South Carolina, 921 Assembly Street, Columbia, SC 29208, USA; 2 Department of Environmental Health Sciences, Arnold School of Public Health, University of South Carolina, Columbia, SC, USA; 3 South Carolina Cancer Prevention and Control Program, University of South Carolina, Columbia, SC, USA; 4 Department of Epidemiology, Colorado School of Public Health, University of Colorado Denver; Aurora, CO, USA; 5 Division of Biostatistics & Epidemiology, Department of Medicine, Medical University of South Carolina, Charleston, SC, USA; 6 Wake Forest University School of Medicine, Winston-Salem, NC, USA; 7 University of North Carolina at Chapel Hill, Gillings School of Global Public Health and School of Medicine, Chapel Hill, NC, USA

**Keywords:** Socioeconomic status, Type 1 diabetes, Risk factors, Youth

## Abstract

**Background:**

European ecologic studies suggest higher socioeconomic status is associated with higher incidence of type 1 diabetes. Using data from a case-control study of diabetes among racially/ethnically diverse youth in the United States (U.S.), we aimed to evaluate the independent impact of neighborhood characteristics on type 1 diabetes risk. Data were available for 507 youth with type 1 diabetes and 208 healthy controls aged 10-22 years recruited in South Carolina and Colorado in 2003-2006. Home addresses were used to identify Census tracts of residence. Neighborhood-level variables were obtained from 2000 U.S. Census. Multivariate generalized linear mixed models were applied.

**Results:**

Controlling for individual risk factors (age, gender, race/ethnicity, infant feeding, birth weight, maternal age, number of household residents, parental education, income, state), higher neighborhood household income (p = 0.005), proportion of population in managerial jobs (p = 0.02), with at least high school education (p = 0.005), working outside the county (p = 0.04) and vehicle ownership (p = 0.03) were each independently associated with increased odds of type 1 diabetes. Conversely, higher percent minority population (p = 0.0003), income from social security (p = 0.002), proportion of crowded households (0.0497) and poverty (p = 0.008) were associated with a decreased odds.

**Conclusions:**

Our study suggests that neighborhood characteristics related to greater affluence, occupation, and education are associated with higher type 1 diabetes risk. Further research is needed to understand mechanisms underlying the influence of neighborhood context.

## Background

Although type 1 diabetes mellitus is one of the leading chronic diseases of childhood and youth, little is known about its causes. Type 1 diabetes has been previously known as insulin-dependent or juvenile-onset diabetes. It results from the destruction of pancreatic beta cells which leads to insulin deficiency and lifelong dependency on insulin therapy. Type 1 diabetes risk is influenced by both genetic and environmental risk factors, but the rapid worldwide increase in incidence suggests that strong environmental influences interact with a common genetic risk set [1,2]. A multitude of environmental risk factors are being studied, including infectious agents, environmental toxins in water or foods, dietary exposures, and exposures to immunizations and pets [3,4]. The Environmental Determinants of Diabetes in Youth (TEDDY) study, a large, international cohort project, was explicitly designed to explore these and other factors with respect to their impact on the development of islet cell autoimmunity and the incidence of type 1 diabetes among high-risk newborns with specific human leukocyte antigen (HLA) genotypes [5]. Because key TEDDY results will not be forthcoming for several years, evaluation of existing studies may prove informative.

A substantial body of research has described marked geographic variation of type 1 diabetes incidence at the ecologic or aggregate level. In Europe, numerous studies have evaluated the association of neighborhood deprivation, income levels, household crowding, population density and urbanization with type 1 diabetes incidence [6]. Only few studies to date have explored these questions in North America [6]. However, associations between incidence rates and neighborhood socioeconomic characteristics observed at the aggregate level do not necessarily apply to individuals. To the best of our knowledge, no individual-level studies of neighborhood-level socioeconomic factors in relation to type 1 diabetes have been published.

To advance the understanding of the impact of neighborhood-level socioeconomic characteristics on risk of type 1 diabetes, we analyzed data from the SEARCH Case-Control (SEARCH CC) study, a recently completed population-based study of ethnically and geographically diverse youth with diabetes in the United States (U.S.). In the context of the Spatial Epidemiology of Diabetes project [7] we extended the SEARCH CC data to include geospatial data and information on Census tract-level characteristics obtained from the U.S. Bureau of the Census. We used the Census tract as a surrogate measure of a person's neighborhood and will therefore refer to the Census tract characteristics as neighborhood characteristics.

## Methods

### Study design and data collection

SEARCH for Diabetes in Youth is a multi-center study that began conducting population-based ascertainment of non-gestational cases of diagnosed diabetes in youth less than 20 years of age in 2001 for prevalent cases and continues with ascertainment of incident cases through the present in five study centers. Details of the SEARCH study methods have been published [8]. In brief, using Health Insurance Portability and Accountability Act compliant procedures, youth with diabetes identified by the SEARCH surveillance effort were asked to complete a brief survey and were then invited to the SEARCH study visit which involved questionnaires, a brief physical examination and laboratory measurements. Ascertainment was conducted using a network of health care providers including pediatric endocrinologists, hospitals, and other providers. Case reports were validated through physician reports, medical record reviews, or in a few instances, self-report of a physician's diagnosis of diabetes [8]. Diabetes type, as assigned by the health care provider, was categorized as type 1, type 2, and other type (including hybrid type, maturity onset of diabetes in youth, type designated as "other", type unknown by the reporting source, and missing). Type 1 diabetes, previously termed insulin-dependent diabetes or juvenile-onset diabetes, subsumes type 1a (immune-mediated diabetes, due to autoimmune destruction of the pancreatic beta cells, leading to absolute insulin deficiency), and type 1b (idiopathic diabetes, of unknown etiology, with varying levels of insulin deficiency). Our analyses here are limited exclusively to youth with type 1 diabetes. Cases of type 2 diabetes, previously referred to as non-insulin-dependent diabetes, were excluded because type 2, which encompasses individuals who are insulin resistant and usually have relative (rather than absolute) insulin deficiency, is thought to have a very different etiology from type 1 diabetes.

**Figure 1:**
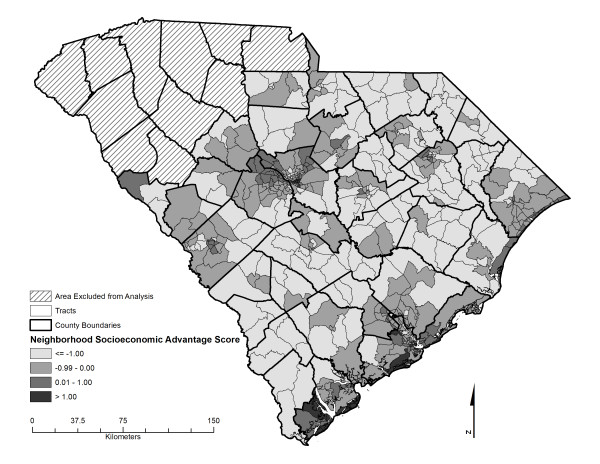
**Neighborhood socioeconomic advantage score characteristics in South Carolina study area**.

**Figure 2:**
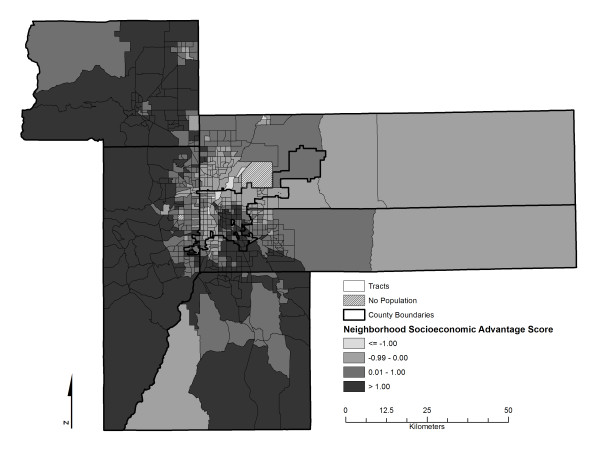
**Neighborhood socioeconomic advantage score characteristics in Denver, Colorado, metropolitan study area**.

SEARCH CC is an ancillary study to SEARCH, conducted at two of the six SEARCH clinical study centers between 2003 and 2006, including the Colorado and South Carolina study centers. For the purposes of the SEARCH CC study, eligibility of cases was restricted to (1) 4 counties surrounding the city of Columbia, including Richland, Lexington, Orangeburg, Calhoun) in South Carolina for 2001 prevalent cases and statewide in subsequent years for incident cases, and (2) selected counties in Colorado (6 counties encompassing the Denver metropolitan area, including Adams, Arapahoe, Boulder, Denver, Douglas, Jefferson, and Weld counties) for 2001 prevalent cases and incident cases and. The study areas are depicted in Figures 1 and 2.

Recruitment of SEARCH CC cases and controls occurred between July 2003 and March 2006. All SEARCH participants seen during this time period who were aged 10 or older were invited to participate in the case-control study protocol. Because type 1 diabetes manifests in severe symptoms after onset and is rapidly fatal if not treated with insulin, all incident cases of type 1 diabetes rapidly seek medical care. Thus, for this particular disease, reliance on health care providers for ascertainment does not introduce an opportunity of selection bias associated with health care access. Controls were concurrently recruited from primary care offices, following the rationale that all SEARCH cases arose from health care provider offices. Participating primary care offices provided an initial study brochure, and patients and their parent or guardian were asked to complete a one-page information form and an indication of permission for study staff to contact them. Of 1,203 information forms returned by participating practices, 881 (73.2%) indicated interest in learning about the study, of whom 41 were ineligible, 233 later refused explicitly, 389 could not be successfully contacted ("passive refusals"), and 218 participated as controls in SEARCH CC. All controls were confirmed as not having diabetes by fasting glucose values obtained during the visit. However, because no primary care providers were selected as control recruitment sites in the upstate area of South Carolina (this includes the counties of Abbeville, Anderson, Cherokee, Greenville, Greenwood, Laurens, Oconee, Pickens, Spartanburg, Union, and York), we decided to exclude any cases originating in the Upstate for the purposes of these case control analyses.

During the study visit, information was collected from the biological mother of cases and controls on date of birth, gender, race/ethnicity, parental education, household family size, household income, maternal age at birth of participant, birth order, birth weight, and infant feeding (including duration of breast feeding and timing of introduction of formula and other foods and beverages). Recall of infant feeding has been shown to be remarkably accurate, even after many years [9,10].

### Geocoding and geo-spatial allocation

The contact addresses provided by SEARCH CC participants were used to identify the Census tract of primary residence, the details of which have been described [11]. Geocoding was conducted in a standardized manner by a single staff person (JDH) traveling to both centers and using ArcGIS 9.3 software (Environmental Systems Research Institute, Redlands, CA 2008) and the TIGER 2000 Road Network File complemented with Zip Code Tabulation Areas data [12]. The vast majority of addresses were geocoded to the street address level (overall 98.7% cases and 95.7% controls in South Carolina, 98.8% of cases and 99.2% of controls in Colorado) and could thereby be allocated to a Census tract to align with Census data (see below). There remained seven cases and five controls that were geocodable to a zip code only. These were allocated by a geo-imputation method [11] which assigns the non-geocodable address to a Census tract within the boundaries of the known zip code based on a random assignment distribution process, thereby avoiding spurious spatial clustering associated with other methods. We have previously shown that this process yields a distribution of cases across tracts that best mirrors the true distribution [11].

The South Carolina study area contained a total of 575 Census tracts and the Colorado area 619 tracts. Given that diabetes in youth is a rare condition and our study included only a small number of years of incidence, it was not surprising that only 13% of Census tracts in the South Carolina area housed one or more cases, and 39% of the Colorado tracts. With respect to controls, 9% of tracts contained one or more control in South Carolina and 14% of Colorado tracts.

### Demographic and socioeconomic Census tract characteristics

Census tract data were obtained from Summary File 1, 2, and 3 from the U.S. Bureau of the Census for 2000 [13]. These data included tract area and a variety of population estimates including total population, race/ethnic-specific population, number working outside the county, number with high school and above education, number unemployed, number living below poverty, number receiving social security, and the number employed in managerial positions. Furthermore, we obtained estimates of the number of households receiving interest, dividend and net rental income, number of housing units, housing units with vehicles, housing units with greater than one person per room, median household income, and median value of housing. Raw data were used to calculate the appropriate estimates of the percent of population with a specific attribute. These were used as a continuous measure in most analyses.

Using a previously developed methodology [14], we also created an area-level composite score of neighborhood socioeconomic status, utilizing Census tract-level information from the 2000 US Census. In the first step, we applied factor analyses, a data reduction technique, to a large set of Census tract socioeconomic indicators and identified a primary factor on which four key variables loaded. These included (1) percent of households with income derived from interest, dividend and rental sources, (2) median value of housing of owner occupied housing units, (3) percent of population with college education or more, and (4) percent of population in managerial positions. In a subsequent step, the summary score was created, the neighborhood socioeconomic advantage score, by summing the Z-scores of the aforementioned four variables, [14]. Note, increasing values represent increasing socioeconomic advantage.

Rural-Urban Commuting Areas (RUCAs) have been developed to characterize the Census tracts in the U.S. with respect to their rural and urban status [15]. RUCAs are based on the U.S. Census Bureaus definitions of urbanized areas and urban clusters, in conjunction with information on work commuting patterns. We converted the ten-tiered RUCA codes developed by the ERS/USDA [15] into a four-tiered system as recommended by using only the primary and secondary RUCA codes [16], thereby differentiating urban core from sub-urban areas, large rural towns, and small towns/isolated rural areas.

### Final Subject Inclusion and Exclusion

The SEARCH CC study included 780 participants (565 cases, 215 controls) from South Carolina and Colorado. Of these, 12 participants (6 from each study center) were removed because of entirely missing address information. Furthermore, 59 participants (53 cases, 6 controls) were removed from the upstate region of South Carolina because an inadequate number of controls were recruited in the region, resulting in our final sample of 709 participants (505 cases, 204 controls).

### Statistical Analysis

We used SAS software version 9.2 (Cary, NC) for all analyses. Descriptive statistics obtained include frequency distributions and means and standard deviations by study center and case status. The proportion of missing data on variables considered potential confounders was as follows: people in household 0.3%, maternal age 12.5%, age fed food other than breast milk 17.8, age at introduction of solid foods 16.2%, and birth weight 13.7%. Thus, the multiple imputation (MI) procedure in SAS was used to perform imputations on these variables resulting in three imputed datasets.

Generalized linear mixed model analyses (PROC GLIMMIX) were used to fit logistic regression models for dichotomous responses (case, control) on the three imputed datasets assuming a binomial distribution and a logit link function. The intercept was specified as a random effect assuming each Census tract has a different intercept. The results of the three imputed datasets were combined and analyzed by using the MIANALYZE procedure. Unadjusted odds ratios (ORs) and adjusted ORs with 95% confidence intervals (95% CI) are reported.

We present here the results of a sequential modeling process to evaluate the relationship between each neighborhood characteristics and the risk of type 1 diabetes, in which each relationship was estimated four times: First, we tested separately the main exposure variables (neighborhood risk factors) without potential confounders. Subsequently, race/ethnicity was added as a first level of adjustment, followed by additional individual-level demographic, socioeconomic and perinatal and infant feeding variables (i.e. gender, age, education, income, number of people living in the household, maternal age, age fed food other than breast milk, age of first solid food introduction, birth weight and study center). In a final level of adjustment, we added number of siblings and birth order.

## Results

Characteristics of SEARCH CC study participants are shown in Table 1 by case status, first for the entire study, and then stratified by study center. The average age was about 14.7 years. Cases and controls differed significantly on several characteristics, including gender (a higher percentage of controls being female), race/ethnicity (a higher proportion of controls being of minority race/ethnicity), education (a lower proportion of controls having more highly educated parents) and income (a lower proportion of controls having higher levels of household income). Cases and controls did not differ with respect to any of the perinatal or infant feeding attributes except the number of siblings.

**Table 1:** Individual level characteristics of cases with type 1 diabetes and controls, SEARCH CC Study (n = 709)

**Individual** **Characteristics**	**Colorado and South Carolina Combined**			**Colorado**			**South Carolina**		
			
	**Cases** **(n = 505)**	**Controls** **(n = 204)**	**p-value**	**Cases** **(n = 411)**	**Controls** **(n = 124)**	**p-value**	**Cases** **(n = 94)**	**Controls** **(n = 80)**	**p-value**
	
Age at visit, mean (SD)	14.8 (3.5)	14.6 (2.9)	0.3086	15.0 (3.4)	14.5 (2.9)	0.0633	14.0 (3.7)	14.7 (2.9)	0.1170
People in house, mean (SD)	4.1 (1.3)	4.1 (1.3)	0.9763	4.1 (1.2)	4.2 (1.2)	0.4112	4.4 (1.7)	4.0 (1.3)	0.1592
Maternal age, mean (SD)	28.7 (5.5)	27.9 (5.8)	0.1108	28.9 (5.5)	28.2 (6.0)	0.2969	27.9 (5.5)	27.4 (5.5)	0.5536
Age fed other than breast milk (days), mean (SD)	80.7 (83.5)	72.1 (78.6)	0.2327	83.7 (79.2)	97.5 (80.1)	0.1155	67.1 (100.8)	31.3 (56.0)	0.0106
Age introduced solid food (days), mean (SD)	169.5 (93.1)	185.7 (147.9)	0.1715	163.2 (81.2)	199.1 (175.3)	0.0385	197.2 (130.3)	165.0 (87.6)	0.0786
Birth weight (ounces), mean (SD)	119.0 (20.8)	115.8 (21.9)	0.0872	118.7 (20.3)	117.3 (21.8)	0.5305	120.4 (23.0)	113.5 (22.1)	0.0596
Siblings, mean (SD)	2.0 (1.3)	2.3 (1.5)	0.0050	1.9 (1.1)	2.2 (1.2)	0.0303	2.4 (1.9)	2.6 (1.8)	0.5502
Birth order, mean (SD)	1.9 (1.0)	1.9 (0.9)	0.7875	1.8 (1.0)	1.8 (0.9)	0.9306	2.0 (1.1)	1.9 (0.9)	0.7885
Race/ethnicity, n (%)									
African American/Hispanic	101 (20.0)	91 (44.6)	< 0.0001	64 (15.6)	41 (33.1)	< 0.0001	37 (39.4)	50 (62.5)	0.0023
Non-Hispanic White	404 (80.0)	113 (55.4)		347 (84.4)	83 (67.0)		57 (60.6)	30 (37.5)	
Gender, n (%)									
Female	250 (49.5)	124 (60.8)	0.0065	199 (48.4)	75 (60.5)	0.0185	51 (54.3)	49 (61.3)	0.3523
Male	255 (50.5)	80 (39.2)		212 (51.6)	49 (39.5)		43 (45.7)	31 (38.7)	
Education*, n (%)									
High school or less	75 (14.9)	54 (26.7)	0.0002	50 (12.2)	21 (17.1)	0.6656	25 (26.6)	33 (41.8)	0.0352
More than high school	428 (85.1)	148 (73.3)		359 (87.9)	102 (82.9)		69 (73.4)	46 (58.2)	
Income*, mean (SD)									
< $ 24,999	59 (13.0)	47 (25.3)	< 0.0001	31 (7.3)	14 (11.5)	0.1016	28 (31.0)	33 (41.2)	0.2969
$ 25,000-74,999	180 (39.6)	84 (45.2)		147 (42.8)	49 (50.8)		33 (51.0)	35 (47.5)	
> $ 75,000	215 (47.4)	55 (29.6)		199 (49.9)	46 (37.7)		16(18.0)	9 (11.2)	

*parental attribute

The geographic study areas are depicted in Figures 1 and 2. These maps additionally illustrate the geographic distribution of the neighborhood socioeconomic advantage score across the South Carolina and Colorado study areas. Marked and statistically significant difference in levels of neighborhood characteristics were observed between type 1 diabetes cases and controls (Table 2). Compared to controls, type 1 diabetes cases lived in neighborhoods with lower levels of unemployment, poverty, household crowding, social security recipients and a smaller proportion of residents of minority race/ethnicity. Furthermore, case participants' neighborhoods exhibited higher median housing values, a higher proportion of the population working outside of the county, higher percent income from interest, higher percent of the population in managerial positions, or with high school education or above, and higher vehicle ownership. Likewise, the neighborhood socioeconomic advantage score indicated higher levels of wealth in type 1 diabetes neighborhoods. In terms of rurality, cases tended to reside more frequently in suburban neighborhoods than controls.

**Table 2:** Neighborhood level characteristics of cases with type 1 diabetes and controls, SEARCH CC Study (n = 709)

Census-tract Characteristics	Colorado and South Carolina Combined			Colorado			South Carolina		
			
	Cases (n = 505)	Controls (n = 204)	p-value	Cases (n = 411)	Controls (n = 124)	p-value	Cases (n = 94)	Controls (n = 80)	p-value
Unemployment (%)	2.5 (1.8)	3.2 (2.2)	0.0003	2.3 (1.8)	2.6 (1.7)	0.1357	3.4 (1.8)	4.0 (2.5)	0.0542
Population below poverty (%)	6.9 (7.4)	11.1 (10.4)	< 0.0001	5.3 (6.0)	7.4 (6.8)	0.0023	13.6 (9.3)	16.8 (12.2)	0.0599
Household crowding (%)	3.4 (4.6)	4.6 (5.0)	0.0040	3.5 (5.0)	4.9 (6.1)	0.0221	3.2 (2.2)	4.2 (2.7)	0.0105
Social security recipient (%)	17.5 (10.1)	22.0 (9.4)	< 0.0001	16.2 (10.0)	19.1 (9.3)	0.0031	23.4 (8.8)	26.5 (7.9)	0.0166
Minority population (%)	23.7 (19.7)	36.5 (27.9)	< 0.0001	20.6 (17.6)	28.1 (22.3)	0.0008	37.3 (22.6)	49.5 (30.7)	0.0037
Homeowners (%)	76.5 (19.6)	71.1 (20.0)	0.0010	77.8 (19.2)	72.4 (21.2)	0.0112	70.7 (20.5)	69.0 (18.0)	0.5523
Median housing value ($)	184K (96K)	144K (74K)	< 0.0001	206K (93K)	183K (67K)	0.0028	88K (32K)	84K (31K)	0.3877
Working outside county (%)	45.0 (19.0)	38.2 (18.9)	< 0.0001	48.7 (16.9)	48.6 (10.1)	0.9354	29.0 (19.9)	22.2 (18.2)	0.0196
Income from interest and others (%)	43.3 (16.3)	35.2 (17.2)	< 0.0001	47.2 (14.7)	42.4 (15.7)	0.0030	26.4 (11.2)	24.0 (13.0)	0.1894
Management position (%)	40.5 (14.3)	33.9 (14.0)	< 0.0001	42.8 (13.9)	37.8 (14.3)	0.0007	30.1 (10.7)	27.8 (11.2)	0.1722
High school education and above (%)	88.9 (11.4)	82.7 (13.6)	< 0.0001	90.8 (10.7)	85.9 (13.6)	0.0003	80.4 (10.8)	77.7 (12.0)	0.1201
Median household income ($)	61K (26K)	49K (20K)	< 0.0001	66K (25K)	57K (19K)	< 0.0001	39K (12K)	36K (12K)	0.0725
Vehicle ownership (%)	95.3 (5.6)	92.1 (8.9)	< 0.0001	96.1 (4.6)	94.3 (5.8)	0.0017	91.9 (8.0)	88.6 (11.5)	0.0360
Neighborhood socioeconomic advantage score	0.5 (0.9)	0.04 (0.9)	< 0.0001	0.8 (0.8)	0.5(0.8)	0.0016	-0.5 (0.6)	-0.7 (0.6)	0.1327
Urban category n (%)									
Small town	6 (1.2)	3 (1.5)	0.0141	2	0	0.7791	4 (4.0)	3 (3.7)	0.0285
Large town	15 (3.0)	16 (7.8)		1	0		14 (16.0)	16 (20.0)	
Suburban	59 (11.7)	15 (7.4)		38 (8.6)	10 (6.6)		21 (23.0)	5 (6.2)	
Urban core	425 (84.2)	170 (83.3)		370 (91.4)	114 (93.4)		55 (57.0)	56 (70.0)	

We subsequently evaluated the independent association of each neighborhood characteristic with odds of type 1 diabetes (Table 3). The first level of adjustment for individual race/ethnicity attenuated but did not explain most of the associations. A one standard deviation increase in the neighborhood socioeconomic advantage score was associated with a 47% increase in odds of type 1 diabetes. Adjusting for additional individual characteristics such as demographic, socioeconomic, perinatal and infant feeding characteristics (adjustment 2) further attenuated some of the associations, including the neighborhood score. However, evidence for an independent effect of several neighborhood-level influences remained: percent population living in poverty (p = 0.008), household crowding (p = 0.0497), social security recipients (p = 0.002), and minority population (p = 0.0003) were significantly associated with a reduced odds of type 1 diabetes, while increasing median household income (p = 0.005), vehicle ownership (p = 0.03), high school education (0.005), percent working outside the county (p = 0.04) and managerial job positions (p = 0.02) were significantly associated with an increased odds of type 1 diabetes. When we additionally adjusted for the individual characteristics of number of siblings and birth order (adjustment 3), the results remained virtually unchanged, although the impact of neighborhood population in managerial positions, household crowding, and working outside the county was slightly attenuated and lost statistical significance. When analyses were stratified by study center, the magnitude of associations was almost identical for Colorado and South Carolina (data not shown). Across the analyses shown in Table 3, the estimated variance of the Census tract random intercept ranged from 0.0081 to 3.4225. The random intercept term was not significant in any of the models, with the one exception being the model of vehicle ownership using adjustment 2, which suggests that there is no consistent evidence for an exclusively spatial effect of diabetes risk associated with the Census tracts.

**Table 3:** Associations of neighborhood characteristics with odds of type 1 diabetes in combined Colorado and South Carolina sample

	Colorado and South Carolina							
	
	Unadjusted		Adjustment 1		Adjustment 2		Adjustment 3	
Neighborhood characteristics	OR	95% CI	OR	95% CI	OR	95% CI	OR	95% CI
Unemployment (+5%)	**0.44**	**(0.28,0.68)**	0.69	(0.45, 1.06)	0.71	(0.46, 1.10)	0.83	(0.52,1.32)
Population below poverty (+5%)	**0.76**	**(0.69,0.84)**	**0.84**	**(0.76,0.93)**	**0.86**	**(0.76,0.96)**	**0.88**	**(0.78,0.99)**
Household crowding (+5%)	**0.79**	**(0.67,0.92)**	0.96	(0.80,1.14)	**0.83**	**(0.69,1.00)**	0.91	(0.75,1.11)
Social security recipient (+5%)	**0.81**	**(0.74,0.88)**	**0.83**	**(0.76,0.90)**	**0.87**	**(0.79,0.95)**	**0.85**	**(0.77,0.93)**
Minority population (+5%)	**0.89**	**(0.86,0.92)**	**0.93**	**(0.89,0.97)**	**0.93**	**(0.89,0.97)**	0.94	(0.90,0.99)
Homeowners (+5%)	**1.07**	**(1.03,1.11)**	1.04	(1.00,1.08)	1.04	(1.00,1.09)	1.03	(0.98,1.07)
Median housing value (+ $10,000)	**1.07**	**(1.04,1.09)**	**1.04**	**(1.02,1.07)**	1.02	(0.99,1.06)	1.02	(0.99,1.05)
Working outside county (+5%)	**1.10**	**(1.05,1.14)**	**1.07**	**(1.02,1.12)**	**1.06**	**(1.00,1.12)**	1.05	(0.99,1.11)
Income from interest and others (+5%)	**1.16**	**(1.10,1.22)**	**1.09**	**(1.03,1.16)**	1.06	(0.99,1.14)	1.03	(0.96,1.10)
Management position (+5%)	**1.18**	**(1.11,1.25)**	**1.11**	**(1.04,1.18)**	**1.09**	**(1.01,1.17)**	1.07	(0.99,1.15)
High school education and above (+5%)	**1.21**	**(1.13,1.29)**	**1.13**	**(1.05,1.21)**	**1.12**	**(1.04,1.21)**	**1.09**	**(1.01,1.19)**
Median household income (+ $10,000)	**1.29**	**(1.19,1.40)**	**1.21**	**(1.11,1.32)**	**1.14**	**(1.03,1.27)**	**1.14**	**(1.03,1.27)**
Vehicle ownership (+5%)	**1.40**	**(1.23,1.59)**	**1.26**	**(1.10,1.44)**	**1.08**	**(1.02,1.17)**	**1.18**	**(1.02,1.37)**
Neighborhood socioeconomic advantage score	**1.79**	**(1.47,2.17)**	**1.47**	**(1.19,1.82)**	**1.25**	**(0.95-1.65)**	**1.32**	**(0.99,1.77)**

Adjustment 1: Race/ethnicity

Adjustment 2: Race/ethnicity, gender, age, education, income, persons living in household, maternal age, age fed food other than breast milk, age at solid food introduction, birth weight, and study center

Adjustment 3: Variables included in adjustment 2 plus number of siblings and birth order

Data presented in bold are statistically significant at p < 0.05.

Because of the case-control differences in the proportion of minorities, we additionally stratified by race/ethnicity (517 non-Hispanic white, 192 minority youth). Fewer associations were statistically significant, but the magnitude of associations between neighborhood characteristics and type 1 diabetes was virtually identical in white and non-white youth (data not shown).

## Discussion

To the best of our knowledge, this report is the only case-control study of type 1 diabetes to date that has focused on neighborhood socioeconomic characteristics. We found marked associations of a large number of Census-based measures of neighborhood socioeconomic status with risk of type 1 diabetes, independent of individual-level covariates. Specifically, attributes related to lower socioeconomic status such as poverty and social security income were associated with lower odds of type 1 diabetes. Consistent with these indicators, the percent minority population, which is frequently related to lower socioeconomic status in the US [17], was also associated with lower type 1 diabetes risk. Conversely, measures of higher socioeconomic status, including educational level, household income, managerial position, vehicle ownership, and working outside of the country, were associated with higher odds of type 1 diabetes. Overall, our results are characterized by consistency of the magnitude and the direction of effect estimates.

While research on socioeconomic patterning of type 1 diabetes risk in Europe is abundant, few studies have been conducted in North America [6]. These included the Jefferson County, Alabama, and the Pittsburg registry [18,19]. The Chicago Childhood Diabetes Registry has repeatedly reported on socioeconomic status and type 1 diabetes incidence, but with somewhat inconsistent results [20,21]. In an earlier publication, neighborhood income, educational level and dwelling size were positively associated with increased rates of type 1 diabetes in African American but not in Hispanic youth. No data were shown for non-Hispanic white youth [20]. More recently, this group studied the impact of changes in neighborhood socioeconomic status over time [21]. Neighborhoods experiencing a change towards lower income levels seemed to be observing lower rates of type 1 diabetes compared to socioeconomically stable neighborhoods. Contrary to expectations, however, emerging high-income neighborhoods were also associated with lower rates of type 1 diabetes [21]. Finally, the Montreal registry and the multi-center SEARCH for Diabetes in Youth study have both found higher incidence rates of type 1 diabetes associated with increased neighborhood wealth [22,23].

Our study differs in a number of ways from previous work. Unlike the ecologic studies discussed above, the case-control design of our study allowed us to make inferences about individual-level risk factors. The study area comprised a markedly larger and more demographically varied area than any previous effort in the US. We included both Colorado and South Carolina residents from neighborhoods across the entire spectrum of socioeconomic status and population density. A recent, very large, hospital-record based case-control study conducted in Washington state found that multiple individual measures of lower socioeconomic status, such as having Medicaid insurance, an unmarried mother or inadequate prenatal care, were associated with decreased odds of type 1 diabetes [24]. Similar to our own work, the study by D'Angeli et al. [24] controlled for a wide array of individual-level covariates. It did not, however, consider the influence of neighborhood characteristics. Thus, we believe our study is an important link between previous work and future results of ongoing investigations.

Several limitations and strengths of our study are worth mentioning. The address data used to create geo-spatial assignments was based on the residence address provided by the participants upon recruitment but did not include duration of residence at this location. We did not have data on day care attendance, consumption of high nitrosamine foods or cod liver oil, all of which have been associated with type 1 diabetes [25-27] and may well be associated with neighborhood socioeconomic status. It is conceivable that neighborhood effects associated with participation could have biased the results of our case-control analyses, though it has been shown that these effects, if present, are likely small [28,29]. Furthermore, there is a small temporal mismatch between data on neighborhood socioeconomic characteristics used from the US Census 2000 compared to the cases of diabetes occurring between 2001 and 2006. Lastly, due to the need for geo-imputation to Census tract for a small fraction of our study sample we can not exclude the possibility of having introduced some error. On the other hand, strengths of our study include the use of a random intercept model which has been suggested to be less likely to be biased than classical regression models [30]. Furthermore, the geographic and race/ethnic diversity of our study population may have provided our study with sufficient exposure variability to discern associations between neighborhood characteristics and odds of type 1 diabetes. Lastly, our study was a population-based case-control study.

How consistent are the observed associations of neighborhood wealth and higher socioeconomic status with current hypotheses on type 1 diabetes etiology? Of the key causal domains that have been explored, only the hygiene hypothesis is consistent with higher socioeconomic status being a risk factor for type 1 diabetes [31]. It suggests that lack of exposure to early childhood bacterial or viral infections leads to a modulation of the immune system and increased risk for autoimmune diseases such as type 1 diabetes. It is conceivable that higher socioeconomic status is associated with improved hygiene and - possibly through living conditions characterized by more personal space - leads to decreased exposures to infections. Consistent with this hypothesis is also the finding by many studies, including our own, that a higher number of siblings and lower birth order was significantly and inversely related to type 1 diabetes risk [24]. It has furthermore been shown that children who moved more often had a markedly reduced risk of type 1 diabetes [32]. Even in a highly mobile society such as the US, residential instability is still strongly associated with lower socioeconomic status.

In contrast, none of the other etiologic type 1 diabetes hypotheses seem to be entirely consistent with our findings, and in fact would suggest that populations with lower, but not high socioeconomic status, are more likely to develop type 1 diabetes. For instance, the early infant feeding hypothesis suggests that early exposures to solid foods and decreased duration of breastfeeding are associated with higher type 1 diabetes risk. However, both of these behaviors are commonly seen in low, but not in high socioeconomic status populations. Likewise, exposure to toxins in water and food which have been hypothesized to be associated with increased type 1 diabetes risk would be more likely in socially disadvantaged than in high-socioeconomic populations. Specific HLA genotypes known to increase type 1 diabetes risk have been shown to exhibit substantial geographic variation [33], but do not seem to explain differences in seroconversion to beta cell autoimmunity. Lastly, a multitude of mechanisms have been summed under the overload or accelerator hypothesis [34], which suggests that overload of the pancreatic beta cells early in life makes them more prone to autoimmunity and/or beta cell apoptosis. Maternal and infant overweight, both key factors in the overload hypothesis, are also more common in populations with low than with high socioeconomic status. On the other hand, infants less exposed to early life infections tend to grow faster in both height and weight which may overload the beta-cells. Thus in summary, this line of reasoning would suggest that whatever the causal agents associated with higher socioeconomic status may be, they would likely need to be quite strong, as they would need to counterbalance other risk-inducing influences associated with lower socioeconomic status. In conclusion, we believe that further research is needed to understand the mechanisms by which the neighborhood context exerts an impact on risk of type 1 diabetes.

## Abbreviations

(PROC GLIMMIX): Generalized linear mixed model analyses; (HLA): human leukocyte antigen; (MI): multiple imputation; (ORs): odds ratios; (RUCA): Rural-Urban Commuting Area; (SEARCH CC): SEARCH Case-Control; (TEDDY): The Environmental Determinants of Diabetes in Youth; (U.S.): United States

## Competing interests

The authors declare that they have no competing interests.

## Authors' contributions

ADL developed the idea for this manuscript, outlined the analyses and wrote the manuscript. JDH geocoded the data and conducted GIS-based data management. MN conducted statistical analyses. RCP and ABL provided statistical expertise. RCP, APL, DD, ABL, DEP, DBD'A, EJMD reviewed and edited the manuscript and contributed to the discussion. All authors read and approved the final manuscript.
